# Clinical Presentation of Acute Pulmonary Embolism: Survey of 800 Cases

**DOI:** 10.1371/journal.pone.0030891

**Published:** 2012-02-27

**Authors:** Massimo Miniati, Caterina Cenci, Simonetta Monti, Daniela Poli

**Affiliations:** 1 Dipartimento di Area Critica Medico Chirurgica, Università degli Studi di Firenze, Firenze, Italy; 2 Struttura Operativa Dipartimentale (SOD) Malattie Aterotrombotiche, Azienda Ospedaliero-Universitaria di Careggi, Firenze, Italy; 3 Istituto di Fisiologia Clinica del Consiglio Nazionale delle Ricerche (CNR), Pisa, Italy; 4 Fondazione CNR-Regione Toscana “G. Monasterio”, Pisa, Italy; University of Cologne, Germany

## Abstract

**Background:**

Pulmonary embolism (PE) is a common and potentially fatal disease that is still underdiagnosed. The objective of our study was to reappraise the clinical presentation of PE with emphasis on the identification of the symptoms and signs that prompt the patients to seek medical attention.

**Methodology/Principal Findings:**

We studied 800 patients with PE from two different clinical settings: 440 were recruited in Pisa (Italy) as part of the Prospective Investigative Study of Acute Pulmonary Embolism Diagnosis (PISAPED); 360 were diagnosed with and treated for PE in seven hospitals of central Tuscany, and evaluated at the Atherothrombotic Disorders Unit, Firenze (Italy), shortly after hospital discharge. We interviewed the patients directly using a standardized, self-administered questionnaire originally utilized in the PISAPED. The two samples differed significantly as regards age, proportion of outpatients, prevalence of unprovoked PE, and of active cancer. Sudden onset dyspnea was the most frequent symptom in both samples (81 and 78%), followed by chest pain (56 and 39%), fainting or syncope (26 and 22%), and hemoptysis (7 and 5%). At least one of the above symptoms was reported by 756 (94%) of 800 patients. Isolated symptoms and signs of deep vein thrombosis occurred in 3% of the cases. Only 7 (1%) of 800 patients had no symptoms before PE was diagnosed.

**Conclusions/Significance:**

Most patients with PE feature at least one of four symptoms which, in decreasing order of frequency, are sudden onset dyspnea, chest pain, fainting (or syncope), and hemoptysis. The occurrence of such symptoms, if not explained otherwise, should alert the clinicians to consider PE in differential diagnosis, and order the appropriate objective test.

## Introduction

Pulmonary embolism (PE) is a common but still underdiagnosed condition. In a survey of the relevant literature from 1945 through 2002, PE was unsuspected or undiagnosed ante-mortem in 3268 (84%) of 3876 patients who had PE discovered at autopsy [1]. Remarkably, even in the patients with large or fatal PE at autopsy, the majority (1902 of 2448, or 78%) were never suspected of having the disease during life [1].

It is maintained that PE may escape prompt diagnosis because clinical symptoms and signs are nonspecific. Lack of specificity could be a limitation if we were to diagnose PE on clinical grounds only, but it has no bearing on the issue of raising the suspicion of the disease. This depends very much on the clinician's ability to formulate a diagnostic hypothesis by taking into proper account a number of clinical symptoms and signs. Raising the suspicion is the crucial step in the diagnostic work-up of PE because it allows selecting patients for further objective testing [2].

The present study was undertaken to assess the prevalence of clinical symptoms, signs, and their combination in a large sample of patients with PE from two different clinical settings. We focused on the identification of the symptoms and signs that prompted the patients to seek medical attention. We collected the relevant information by interviewing the patients directly using a standardized, self-administered questionnaire.

## Materials and Methods

### Ethics statement

The study protocol was approved by the ethics committee of the Careggi University Hospital, Firenze (Italy). An informed written consent was obtained from each patient prior to study entry.

### Sample

The study included 800 patients with an established diagnosis of PE. Three-hundred-sixty of them were evaluated consecutively at the Unit of Atherothrombotic Disorders (UAD), Careggi University Hospital, Firenze (Italy), between January 1, 2009 and December 31, 2010, for the following reasons:

(a) to search for inherited thrombophilia; (b) to plan the duration of oral anticoagulant therapy; (c) to assess the extent of perfusion recovery by lung scintigraphy within a year of PE diagnosis; (d) to evaluate the right ventricular function by transthoracic echocardiography at the time of perfusion scintigraphy.

These patients had been diagnosed with and treated for acute PE in seven hospitals of central Tuscany. They were referred to the UAD within 4 weeks after hospital discharge.

The 440 other patients with PE were part of a sample of 1100 consecutive patients with suspected PE, who were enrolled in the Prospective Investigative Study of Acute Pulmonary Embolism Diagnosis (PISAPED) at the Institute of Clinical Physiology, Pisa (Italy), between 1991 and 1999 [3]–[6].

### Collection of clinical data

The 360 patients comprised in the Firenze sample were examined by the authors at the outpatient clinic of the UAD. Care was taken to identify risk factors for PE, and pre-existing diseases which may mimic the clinical presentation of PE. Each patient was invited to complete a self-administered standardized questionnaire including the description of the symptoms experienced, and the time interval between the onset of symptoms and the diagnosis of PE (table 1). The questionnaire is in all similar to that used in the PISAPED [3]–[6].

**Table 1:** Standardized questionnaire.

Please, answer the following questions concerning the symptoms you may have had before the diagnosis of pulmonary embolism (PE) was established.
***No symptoms at all***
Yes
No
***Dyspnea (shortness of breath) during exertion or at rest***
Yes
No
*If yes, how would you describe its onset?*
Sudden (in a matter of hours)
Gradual (over a period of several days or weeks)
***Noctural dyspnea (partly relieved by assuming the seated or semirecumbent position)***
Yes
No
***Chest pain***
Yes
No
*If yes, how would you describe it?*
Precordial or substernal (as an oppression over the anterior chest wall)
Pleuritic (as a stabbing or shooting in the chest, exacerbated by breathing, coughing, sneezing, or even talking)
***Fainting or transitory loss of consciousness***
Yes
No
***Bloody sputum***
Yes
No
***Cough (as a new symptom)***
Yes
No
***High fever (>38°C)***
Yes
No
***Unilateral, painful swelling of the upper or lower extremity***
Yes
No
***Other symptoms***
Please, specify:
***Time interval between onset of symptoms and diagnosis of PE***
Within one day
More than one day (please, specify):
***Your location at the time of symptoms' onset***
Home
Hospital

Every effort was made to retrieve from clinical files the electrocardiograms (ECG) obtained on the day of PE diagnosis. The ECGs were reviewed by a cardiologist who was blinded to the diagnosis. Acute right ventricular (RV) overload was deemed present if one or more of the following abnormalities were identified: S-wave in lead I and Q-wave in lead III each of amplitude >1.5 mm, with T-wave inversion in lead III (S_1_Q_3_T_3_), S-waves in lead I, II, and III each of amplitude >1.5 mm (S_1_S_2_S_3_), T-wave inversion in right precordial leads, transient right bundle branch block, and pseudoinfarction [7]–[8].

The 440 patients with PE included in the PISAPED had been examined by one of twelve chest physicians who took part in the study. All the clinical and laboratory data were recorded by the physicians on a standard form before any further objective testing [3]–[6]. Data on the clinical presentation of PE were retrieved from the PISAPED database, and used for comparison with the clinical data acquired in the 360 other patients. The following paragraphs refer to the procedures used for diagnosing PE, assessing perfusion recovery and right ventricular function in the patients comprised in the Firenze sample.

### Diagnostic criteria for pulmonary embolism

PE diagnosis was established by multidetector computed tomographic angiography (CTA), perfusion lung scintigraphy, or ventilation-perfusion scintigraphy. Angiographic criteria included the identification of an embolus obstructing a vessel or the outline of an embolus within a vessel. Perfusion scans were considered positive for PE if showing segmental (wedge-shaped) perfusion defects [3]. Ventilation-perfusion scans were rated “high-probability” for PE if they featured segmental perfusion defects with normal ventilation [9], [10].

PE was classified as provoked if associated with known risk factors such as recent trauma, bone fracture, major surgery, pregnancy/post-partum, active cancer, use of oral contraceptives, or immobilization for longer than 3 consecutive days. In all other instances, it was considered unprovoked.

### Assessment of residual perfusion defects by lung scintigraphy

We estimated the extent of residual perfusion defects on the lung scans obtained between 6 and 12 months of PE diagnosis. Such estimation was carried out by a nuclear medicine specialist, according to a method validated against pulmonary angiography [11]. Briefly, each lobe is attributed a weight according to regional blood flow as follows: right upper lobe, 0.18; right middle lobe, 0.12; right lower lobe, 0.25; left upper lobe, 0.13; lingula, 0.12; left lower lobe, 0.20. The perfusion of each lobe is estimated visually by means of a five-point score (0, 0.25, 0.5, 0.75, 1) where 0 means “not perfused” and 1 “normally perfused”. Visual estimates of perfusion are based on the combined evaluation of six scintigraphic views (anterior, posterior, both lateral, and both posterior oblique). Each lobar perfusion score is obtained by multiplying the weight assigned to the lobe by the estimated perfusion of that lobe. The overall score is the sum of the perfusion scores of the six lobes, and the percentage of pulmonary vascular obstruction is calculated as: (1–overall perfusion score)×100.

### Transthoracic echocardiography and chest radiography

Transthoracic echocardiography and postero-anterior and lateral chest radiographs were obtained at the time of perfusion lung scanning. Echocardiograms were performed and interpreted by an experienced cardiologist. Measured variables included the end-diastolic right ventricle diameter, the thickness of the right ventricle free wall, and the tricuspid regurgitation velocity (if measurable). The right ventricular wall motion was assessed qualitatively. An end-diastolic right ventricle diameter <26 mm, a wall thickness <7 mm, and a tricuspid regurgitation velocity <2.7 m/s were regarded as normal [12].

Chest radiographs were examined by one of the authors (MM) for the presence of dilatation of the pulmonary artery trunk, and of the right ventricle that are suggestive of chronic thromboembolic pulmonary hypertension (CTEPH) [13].

The patients who featured persistent, bilateral perfusion defects in the lung scans taken between 6 and 12 months of PE diagnosis, were re-evaluated by lung scintigraphy and transthoracic echocardiography at 3-month intervals. If the lung scans remained unchanged over time, and the echocardiograms and chest radiographs were suggestive of CTEPH, right heart catheterization and pulmonary angiograms were obtained. Diagnostic criteria included a mean pulmonary artery pressure >25 mmHg with a mean pulmonary occlusion pressure <15 mmHg, and the presence of multiple lobar, segmental, or subsegmental filling defects on selective pulmonary angiography [14].

### Statistical analysis

Differences between groups were assessed by Fisher's exact test for the categorical variables, and by Mood's median test for the continuous variables. Continuous variables in the text and in the tables are reported as median and interquartile range (IQR). Ninety-five confidence intervals (CI) were calculated according to the binomial distribution with continuity correction. Two-tailed p-values of less than 0.05 were considered statistically significant throughout. The statistical analysis was performed with Stata version 10 (StataCorp, College Station, TX).

## Results

### Patient characteristics

The baseline characteristics of the 440 patients with PE from the PISAPED are given in detail elsewhere [3]–[6]. PE was diagnosed by selective pulmonary angiography in 436 and by autopsy in 4. They are used here for the purpose of comparing the prevalence of clinical symptoms and signs with the 360 patients comprised in the Firenze sample. In the latter group, most of the subjects (90%) were outpatients at the time of PE diagnosis, and nearly 70% had unprovoked PE (table 2). In most cases, multidetector CTA was used as the diagnostic technique (table 2); medical treatment consisted of unfractionated heparin or low molecular weight heparins in 88% of the patients (table 2).

**Table 2:** Baseline characteristics of 360 patients with pulmonary embolism (Firenze sample).

	Number or Median	(Percent or IQR)
*Baseline characteristics*		
Outpatients	324	(90)
Time to diagnosis, days	2	(1–7)
Age, years	61	(46–71)
Male sex	162	(45)
Unprovoked PE	237	(66)
Prior cardiovascular disease	86	(24)
Prior pulmonary disease	20	(6)
Active cancer	20	(6)
*Diagnostic technique*		
MD-CTA	298	(83)
Perfusion lung scintigraphy	56	(15)
Ventilation-Perfusion scintigraphy	6	(2)
*Therapy in the acute stage*		
Unfractionated heparin	227	(63)
Low molecular weight heparins	90	(25)
Fondaparinux	29	(8)
Thrombolysis	14	(4)

IQR = interquartile range. PE = pulmonary embolism. MD-CTA = multidetector computed tomographic angiography.

All the 360 patients completed the scintigraphy follow-up. By one year of diagnosis, the median score of residual perfusion defects was 0% (IQR, 0–10%). Five patients showed persistent, bilateral perfusion defects consistent with chronic PE. Three of them (0.8% of 360) met the hemodynamic criteria of CTEPH. Such incidence is nearly the same as in the PISAPED [15].

### Symptoms and signs (Firenze sample)

The prevalence of clinical symptoms and signs is reported in table 3. They were in decreasing order of frequency: sudden onset dyspnea, chest pain, unilateral painful swelling of the lower or upper extremity, fainting or syncope, and hemoptysis. Very few patients experienced gradual onset dyspnea, cough, or high fever, and none complained of orthopnea. Chest pain was unilateral and pleuritic in type in 118 (84%) of 140 patients. In 17 (94%) of the 18 cases who reported hemoptysis, the symptom was associated with sudden onset dyspnea, chest pain, or both. The median interval between symptoms' onset and diagnosis of PE was 2 days (table 2). Yet, in 25% of the patients, the time to diagnosis exceeded 7 days (median time 20 days). Most of the patients in whom the diagnosis of PE was delayed had sudden unexplained dyspnea as the initial clinical symptom.

**Table 3:** Prevalence of symptoms and signs in 360 patients with pulmonary embolism (Firenze sample).

Symptoms or signs	Number	(%)	(95% CI)
Sudden onset dyspnea	281	(78)	(74–82)
Gradual onset dyspnea	9	(3)	(1–5)
Orthopnea	0	(0)	(0–1)
Chest pain	140	(39)	(34–44)
Fainting or syncope	78	(22)	(18–26)
Hemoptysis	18	(5)	(3–8)
Cough	14	(4)	(2–7)
Unilateral painful swelling of lower or upper extremity	137	(38)	(33–43)
Fever >38°C	15	(4)	(2–7)

CI = confidence interval.

ECGs, obtained on the day of PE diagnosis, were made available in 334 (93%) of 360 patients; signs of acute RV overload were present in 139 of 334 (42%, IQR 36–47%).

### Comparison between the two samples

As shown in table 4, the two samples differed significantly in terms of age, proportion of outpatients at the time of PE diagnosis, prevalence of unprovoked PE, and of active cancer. These differences notwithstanding, the prevalence of symptoms and signs was similar in the two samples. Chest pain prevailed significantly in the PISAPED patients, whereas unilateral swelling of the lower or upper extemity (taken as a sign of deep vein thrombosis [DVT]) was reported more frequently by the patients in the Firenze sample.

**Table 4:** Baseline characteristics and prevalence of clinical findings in 800 patients with pulmonary embolism from two different clinical settings.

	All(n = 800)	Pisa(n = 440)	Firenze(n = 360)	
	%	%	%	P-Value*
Outpatients	52	21	90	<0.001
Age >65 years†	50	58	39	<0.001
Male sex	46	47	45	0.669
Unprovoked PE	51	38	66	<0.001
Active cancer	11	16	6	<0.001
Prior cardiovascular diseases	27	29	24	0.108
Prior pulmonary diseases	7	8	6	0.129
Sudden onset dyspnea	80	81	78	0.251
Gradual onset dyspnea	3	3	3	0.673
Orthopnea	0.4	0.7	0	0.257
Chest pain	49	56	39	<0.001
Fainting or syncope	24	26	22	0.183
Hemoptysis	6	7	5	0.240
Unilateral painful swelling of lower or upper extremity	30	23	38	<0.001
Fever >38°C	5	6	4	0.208
Acute RV overload (ECG)	44	45	42‡	0.306

Data are reported as percent of total in each sample.

RV = right ventricle. ECG = electrocardiogram.

* Pisa versus Firenze.

† Median age in the whole sample of 800 patients.

‡ In 139 of 334 patients in whom ECGs were available.

The prevalence of ECG signs of acute RV overload was nearly identical in the two samples (table 4). Considering the whole sample, the patients with RV overload featured a significantly higher prevalence of sudden onset dyspnea (87% vs 74%, p<0.0001) and of syncope (35% vs 15%, p<0.0001), and a lower prevalence of hemoptysis (3% vs 8%, p = 0.004) than those without RV overload.

### Combination of clinical symptoms and signs in the two samples

At least one of four symptoms (sudden onset dyspnea, chest pain, fainting or syncope, and hemoptysis) were reported by 756 (94%) of 800 patients (table 5). Isolated symptoms and signs of DVT occurred in 22 cases (3%). Twenty had proximal DVT of the lower limb, and two had DVT of the upper limb extending to the subclavian vein. The 22 patients with isolated manifestations of DVT had a median age of 48 years (IQR, 38–60 years), and were significantly younger (p<0.001) than the 778 other patients (median age 66 years, IQR, 53–74 years).

**Table 5:** Combination of clinical symptoms and signs in 800 patients with pulmonary embolism.

	Number	(%)	(95% CI)
Only one of four symptoms*	337	(42)	(39–46)
Any two of four symptoms*	329	(41)	(38–45)
Any three of four symptoms*	90	(11)	(9–14)
At least one of four symptoms*	756	(94)	(93–96)
Other symptoms†	15	(2)	(1–3)
Symptoms and signs of DVT only	22	(3)	(2–4)
No symptoms at all	7	(1)	(0.4–2)

CI = confidence intervals. DVT = deep vein thrombosis.

* Sudden onset dyspnea, chest pain, fainting or syncope, and hemoptysis. These symptoms are not explained otherwise.

† Gradual onset dyspnea (n = 10); palpitations (n = 5).

Only 7 (1%) of 800 patients had no symptoms prior to the diagnosis of PE (table 5). In one, PE was diagnosed incidentally when he was referred unconscious to the radiology department shortly after severe head trauma and multiple bone fractures. The six other patients had minor PE affecting one or two lung segments.

The combination of clinical symptoms and signs are reported separately for the Pisa and Firenze sample in table 6.

**Table 6:** Combination of clinical symptoms and signs in 800 patients with pulmonary embolism (Pisa versus Firenze).

	Pisa (N = 440)		Firenze (N = 360)		
	n	(%)	n	(%)	P-Value*
Only one of four symptoms†	166	(38)	171	(47)	0.006
Any two of four symptoms†	204	(46)	125	(35)	<0.001
Any three of four symptoms†	58	(13)	32	(9)	0.057
At least one of four symptoms†	428	(97)	328	(91)	<0.001
Other symptoms‡	8	(2)	7	(2)	1.000
Symptoms and signs of DVT only	1	(0.3)	21	(6)	<0.001
No symptoms at all	3	(0.7)	4	(1)	0.707

DVT = deep vein thrombosis.

* Pisa versus Firenze.

† Sudden onset dyspnea, chest pain, fainting or syncope, and hemoptysis. These symptoms are not explained otherwise.

‡ Gradual onset dyspnea (n = 10); palpitations (n = 5).

## Discussion

The present study was undertaken to reconsider the clinical presentation of PE with special emphasis on the identification of those symptoms and signs that prompt the patients to seek medical attention. We addressed this issue by interviewing directly the patients using a standardized form that was originally utilized in the PISAPED [3]–[6]. In that study, the patients with suspected PE were examined before they underwent the definitive test to confirm or exclude the diagnosis. The patients included in the Firenze sample could not be interviewed as timely as those in the PISAPED. However, the occurrence of a recall bias seems very unlikely because all of them were evaluated shortly after hospital discharge.

In conformity with the strategy adopted in the PISAPED [3]–[6], [15], all the patients included in the Firenze sample underwent a scintigraphic follow-up to assess the extent of residual perfusion abnormalities between 6 and 12 months of PE diagnosis. Virtually all of them (99%) showed a complete or nearly complete restoration of pulmonary perfusion. So, it seems reasonable to assume that they had had a first episode of acute PE.

The two samples reported on here differ from each other as regards age, proportion of inpatients, prevalence of unprovoked PE and of active cancer. Yet, the prevalence of the reported symptoms and signs is very similar. Sudden unexplained dyspnea was by far the most frequent symptom in both samples, followed by chest pain (usually pleuritic), fainting (or true syncope), and hemoptysis. At least one of the above symptoms was reported by 94% of the patients in the whole sample.

**Figure 1:**
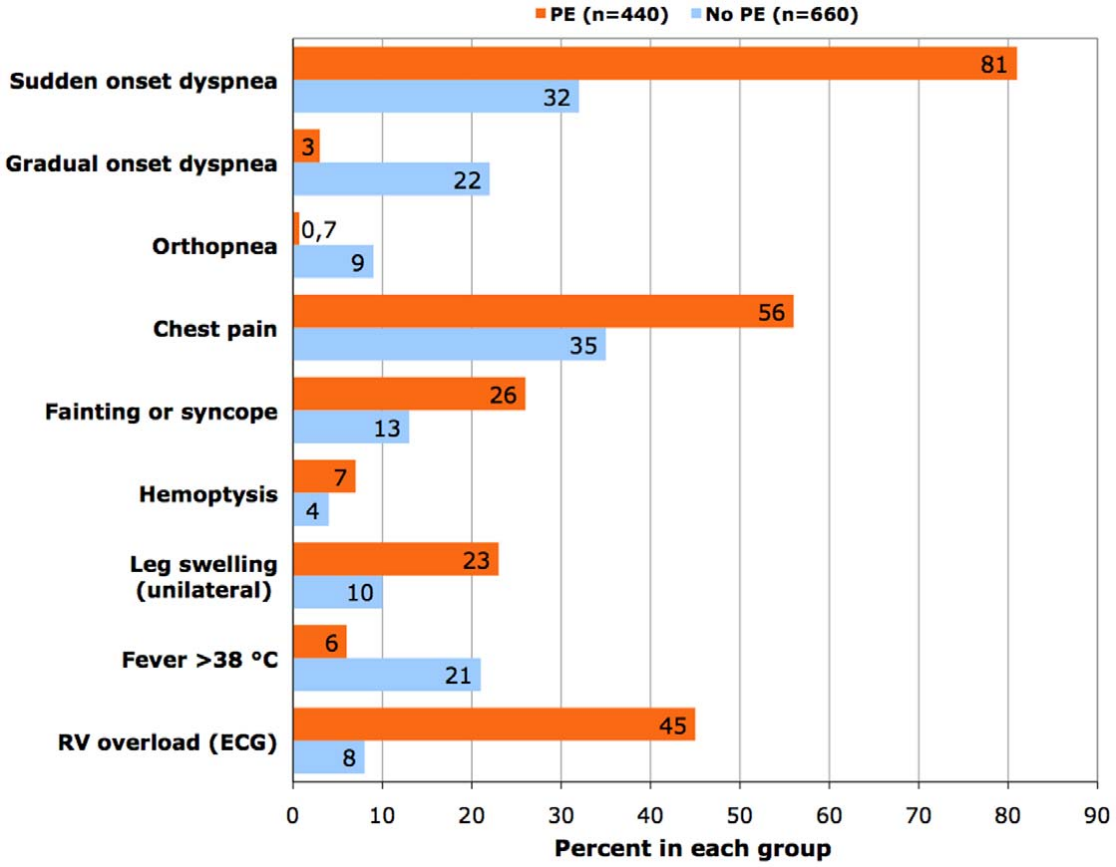
Prevalence of clinical symptoms and signs, and of electrocardiographic (ECG) signs of acute right ventricle (RV) overload in 1100 patients with suspected pulmonary embolism (PE). Data are from reference 5. P-values are <0.001 for all the variables, with the exception of hemoptysis (p<0.05).

In the PISAPED [5], the prevalence of sudden onset dyspnea, chest pain, fainting (or true syncope), and hemoptysis was significantly higher among the 440 patients with PE than in the 660 in whom the diagnosis was excluded (figure 1). Similarly, clinical symptoms and signs suggestive of DVT prevailed significantly in the patients with PE, and so did ECG signs of acute right ventricle overload (figure 1). By contrast, gradual onset dyspnea, orthopnea, and high fever prevailed significantly in the patients in whom PE was ruled out (figure 1). At least one of four symptoms (sudden onset dyspnea, chest pain, fainting/syncope, and hemoptysis) was present in 97% of the 440 patients with PE and in 62% of the 660 without PE (p<0.00001).

In the present study, only 3 out of 800 patients with PE complained of orthopnea. This is at variance with the 36% prevalence of orthopnea reported by Stein et al. in 192 patients with PE enrolled in the PIOPED II [16]. Such remarkable difference is likely the consequence of the criteria used in the two studies to define orthopnea. In the PIOPED II, orthopnea is considered present if the patient is used to lie on two or more pillows, whereas in our study orthopnea is defined as a spell of acute dyspnea (usually, but not necessarily, nocturnal) that forces the patient to assume the seated or semirecumbent position. We preferred such definition because the habit of lying on two or more pillows at night is not unique to left heart failure with pulmonary edema as it may be encountered in chronic obstructive lung disease, asthma, obstructive sleep apnea, and gastro-esophageal reflux.

The prevalence of symptoms and signs suggestive of DVT was significantly higher in the Firenze sample than in the PISAPED. In the latter, however, some 20% of the patients had undergone major abdominal or pelvic surgery before the diagnosis of PE [3]–[6]. So, in these patients, pulmonary emboli may have originated from sites other than the deep veins of the lower limb.

Three percent of the patients presented with symptoms and signs of DVT only. All of them had proximal DVT of the lower or upper extremity, and had PE discovered at pulmonary angiography. Reportedly, about one third of the patients with DVT have “silent” PE, the incidence of the disease being higher with proximal than with distal DVT [17].

Therefore, routine screening for PE seems warranted in the patients with DVT, particularly in those with proximal DVT [17]. Documenting PE in a patient with DVT may justify a more aggressive in-hospital treatment because the short-term survival in patients with PE is much worse that in those with isolated DVT [18].

In our study, 44% of 800 patients with PE had ECG signs of acute RV overload. The occurrence of such abnormalities may strengthen the suspicion of PE in a patient with unexplained abrupt dyspnea, syncope, or chest pain.

We acknowledge that our study has a limitation: it deals with patients in whom the diagnosis of PE was eventually established during life. We can say nothing of those in whom PE was undetected, and who may have died of it. This proportion will probably remain unknown because the rate of autopsies drastically declined over the last 20 years [19]. However, PE is rarely an all-or-none disorder, so it can be timely suspected if due attention is paid to the patient's complaints. In 1967, Felix Fleischner wrote: “…before the acute massive attack, which may prove fatal, there are often telltale warnings that may alert the clinicians to the occurence of minor embolic events” [13]. Our findings are in agreement with this statement.

Raising the suspicion of PE is instrumental to select patients in whom objective testing is needed to confirm or exclude the diagnosis.

Multidetector CTA is now regarded as the first-line imaging technique for suspected PE as it permits the direct visualization of clots in the pulmonary circulation. CT has revolutionized the practice of medicine, particularly in the emergency departments (ED). In a nationwide survey in the United States, the use of CT in the ED rose from 2.7 million in 1995 to 16.2 million in 2007, corresponding to a 5.9-fold increase and an annual growth rate of 16% [20].

Mamlouk el al. evaluated retrospectively the medical records of 2003 consecutive patients (mean age 50 years, inpatients 49%, female 58%) who underwent CTA for possible PE over a 1.5-year period [21]. Inpatients were twice as likely to have PE as those from the ED. Yet, the overall prevalence of PE was of only 9.8% (197/2003). Notably, the occurrence of a positive angiogram in the patients with no risk factors for PE was as low as 1% (5/520).

It seems, therefore, that CTA is increasingly used as a screening method rather than a means to confirm or exclude clinically suspected PE [22]. This may contribute to inflate the costs of the diagnostic procedures, and to expose the patients to an undue amount of radiation. The latter is of concern, especially in women of childbearing age. In fact, using a contemporary 64-detector CTA protocol for PE, the absorbed dose to the female breast is the range of 3.5 to 4.2 cGy [23], which is 30 times as great as that absorbed during ventilation-perfusion scintigraphy (0.08 cGy) [9].

In summary, we found that the most reliable indicator of patients with PE is sudden onset dyspnea. Other symptoms include chest pain, fainting (or syncope), and hemoptysis. The occurrence of such symptoms, if not explained otherwise, should alert the clinicians to consider PE in differential diagnosis. This is the crucial step in the diagnostic work-up of PE. Next, the clinical probability should be assessed, ideally by means of a validated prediction model [4]–[6], [24]. If the clinical probability is low (20% or less), the most practical approach would be to measure the D-dimer concentration by a quantitative assay. If the D-dimer test is negative, PE can be safely ruled out; if positive, additional investigation is required [10]. Should the clinical probability of PE be other than low, it would be sound to order immediately an appropriate imaging technique (multidetector CTA, or lung scintigraphy) to confirm or exclude the diagnosis [10].
